# Pertussis toxin-induced inhibition of Wnt/β-catenin signaling in dendritic cells promotes an autoimmune response in experimental autoimmune uveitis

**DOI:** 10.1186/s12974-023-02707-y

**Published:** 2023-02-04

**Authors:** Zhihui Zhang, Yongtao Li, Nu Chen, Huan Li, Shuang Chen, Xuexue Cui, Hui Shao, Lai Wei, Jianxing Ma, Song Zhang, Xiaorong Li, Xiaomin Zhang

**Affiliations:** 1 Tianjin Key Laboratory of Retinal Functions and Diseases, Tianjin Branch of National Clinical Research Center for Ocular Disease, Eye Institute and School of Optometry, Tianjin Medical University Eye Hospital, Tianjin, China; 2 Department of Ophthalmology and Visual Sciences, Kentucky Lions Eye Center, University of Louisville, School of Medicine, Louisville, KY USA; 3 State Key Laboratory of Ophthalmology, Zhongshan Ophthalmic Center, Sun Yat-sen University, Guangzhou, China; 4 Department of Biochemistry, Wake Forest University School of Medicine, Winston-Salem, NC USA; 5 Institute for Immunology and College of Life Sciences, Nankai University, Tianjin, China

**Keywords:** Pertussis toxin, Dendritic cells, Pathogenic T cell, Experimental autoimmune uveitis

## Abstract

**Background:**

Previous reports have indicated that disrupting the Wnt/β-catenin pathway in dendritic cells (DCs) may affect the progression of autoimmune inflammation; however, the factors and timing that regulate Wnt/β-catenin signaling have not been clearly understood.

**Methods:**

Experimental autoimmune uveitis (EAU) mice and Vogt–Koyanagi–Harada disease (VKH) patient samples were used to detect the expression of Wnt/β-catenin pathway genes. Western blot, real-time PCR, flow cytometry, and ELISA were performed to examine the expression of components of the Wnt/β-catenin pathway and inflammatory factors. DC-specific β-catenin knockout mice and 6-bromoindirubin-3′-oxime (BIO) administered mice were used to observe the effect of disrupting the Wnt pathway on EAU pathogenesis.

**Results:**

Wnt/β-catenin signaling was inhibited in DCs during the induction phase of EAU. The inhibition was mediated by pertussis toxin (PTX), which promoted DC maturation*,* in turn promoting pathogenic T cell proliferation and differentiation. In vivo experiments confirmed that deleting β-catenin in DCs enhanced EAU severity, and pre-injection of PTX advanced EAU onset. Administration of a Wnt activator (BIO) limited the effects of PTX, in turn ameliorating EAU.

**Conclusions:**

Our results demonstrate that PTX plays a key role as a virulence factor in initiating autoimmune inflammation via DCs by inhibiting Wnt/β-catenin signaling in EAU, and highlight the potential mechanism by which infection can trigger apparent autoimmunity.

## Introduction

Autoimmune uveitis is an umbrella term for numerous types of ocular autoimmune diseases that threaten vision in patients of all ages, including Vogt–Koyanagi–Harada disease (VKH), HLA-B27-positive uveitis, sympathetic ophthalmia, and Behçet disease uveitis [1]. However, the etiology of uveitis remains elusive. Based on the findings of animal model studies of experimental autoimmune uveitis (EAU), abnormally activated dendritic cells (DCs) and T helper cell subsets play key roles in EAU pathogenesis [2–5]. Activated DCs regulated by various signaling networks, such as nuclear factor kappa-B (NF-κb), mitogen-activated protein kinase (MAPK), and mammalian target of rapamycin (mTOR) signaling [6–8] control the activation, proliferation, and differentiation of pathogenic T cells by secreting various cytokines. Recent reports have demonstrated that the activation of canonical Wnt/β-catenin signaling programs DCs to a tolerogenic state by regulating the secretion of factors such as interleukin-6 (IL-6), IL-12, and IL-10; that way, Wnt/β-catenin signaling limits the occurrence of autoimmune diseases in clinical settings and in animal models, such as experimental autoimmune encephalomyelitis (EAE), inflammatory bowel disease, and rheumatoid arthritis (RA) [9–12]. Conversely, deletion of β-catenin in DCs promotes the differentiation of CD4^+^ T cell subsets and induces severe autoimmune inflammation [10, 13]. However, the factors and timing that regulate Wnt/β-catenin signaling in DCs have not yet been reported.

Canonical Wnt/β-catenin signaling is required for numerous developmental processes in mutiple organs and species, including cell proliferation, cell fate decisions, cell polarity, and stem cell maintenance [14–17]. The activation of Wnt/β-catenin signaling relies on the binding of Wnt ligands to Frizzled receptors and their co-receptors (LRP5 and LRP6), which results in the stabilization and translocation of cytosolic β-catenin to the nucleus, where it interacts with T cell factor/lymphoid enhancer factor (TCF/LEF) family members to regulate the transcription of target genes [18, 19]. Kallistatin and Dickkopf WNT signaling pathway inhibitor 1 (DKK1) are crucial Wnt antagonists, which serve to inhibit Wnt signaling [20, 21].

Wnt signaling can be regulated by numerous factors, including microbiotas and their toxins. PTX is an A-B toxin produced by the whooping cough-causing bacterium, *Bordetella pertussis* [22]. The A-protomer exhibits ADP-ribosyltransferase activity and ribosylates the α subunits of heterotrimeric Gi/o proteins, resulting in the uncoupling of receptors from the Gi/o proteins. The B-oligomer binds proteins expressed on the cell surface, such as Toll-like receptor 4, and activates an intracellular signal transduction cascade. PTX has been used extensively as an adjuvant for inducing autoimmune disease models, including experimental autoimmune EAE, GPI-induced rheumatoid arthritis (RA), and EAU [23]. The role of PTX in the induction of EAU models had not been completely elucidated. Optimal doses of PTX can promote EAU pathogenesis by increasing vascular permeability, disrupting the blood–eye barrier, and promoting lymphocyte infiltration into the retina; conversely, supraoptimal doses of PTX can prevent EAU progression via the inhibition of lymphocyte recirculation during the efferent phase of the disease [24]. In addition, PTX administration during the lymphocyte migration stage prevents EAU progression by disrupting signaling through PTX-sensitive Gi protein-coupled receptors [25]. Therefore, the dose and timing of PTX treatment influences inflammation severity.

The aim of the present study was to demonstrate the activation status of Wnt/β-catenin signaling in an EAU model and elucidate its effect on EAU pathology. We explore the factors that regulate Wnt/β-catenin signaling in EAU and report that PTX-induced downregulation of Wnt/β-catenin signaling in DCs plays a key role in initiating autoimmune uveitis.

## Materials and methods

### Uveitis patients

Studies involving human participants were reviewed and approved by the Ethics Committee of Tianjin Medical University Eye Hospital, Tianjin, China (No. 2016KY-14). All participants provided written informed consent. Patients with VKH disease were enrolled at Tianjin Medical University Eye Hospital, Tianjin, China. Blood samples were collected from 10 donors diagnosed with VKH and 10 healthy controls for qPCR and FACs. All the patients had newly onset VKH and had not received any therapy. Individuals with comorbidities, including diabetes, hypertension, cancer, and other systemic diseases, were excluded. No remarkable sex or age differences were observed between patients with VKH and healthy controls.

### Animals

All procedures involving mice were approved by the Laboratory Animal Care and Use Committee of Tianjin Medical University Eye Hospital and conformed to the ARVO Statement for the Use of Animals in Ophthalmic and Vision Research. C57BL/6J (B6) mice were obtained from the Vital River Experimental Animal Center (Beijing, China). OT-II TCR transgenic mice were a gift from Prof. Xiaoming Feng (Chinese Academy of Medical Sciences & Peking Union Medical College). β-catenin^flox/flox^ mice (β-catenin^fl/fl^), Axin2 (LacZ) reporter mice, and CD11c-Cre mice were obtained from Jackson Laboratories. β-catenin^flox/flox^ mice were bred with transgenic mice (DC-Cre) expressing Cre enzyme under the control of the CD11c promoter to generate mice lacking β-catenin in DCs (β-cateninDC^−/−^). Mice were maintained under specific pathogen-free conditions.

### EAU induction and clinical evaluation

Mice were immunized subcutaneously on day 0 with 300 µg of Interphotoreceptor Retinoid-Binding Protein (IRBP) 651–670 (Hanheng Group, Shanghai, China) emulsified in CFA (incomplete Freund’s adjuvant plus 4 mg/ml of *Mycobacterium tuberculosis* strain 37RA, Difco). Pertussis toxin (0.5 µg in 200 µl saline; List Biological Laboratories, USA) was administered twice intraperitoneally on day 0 and day 1 after immunization. Disease status was assessed by fundus examination on a scale of 0–4 based on the number, type, and size of lesions and extent of inflammation, as described previously [26].

### Histological evaluation

Mice were euthanized with tribromoethanol on day 21 post-immunization. Eyeballs were obtained from β-catenin^fl/fl^ and β-cateninDC^−/−^ mice and fixed in 10% formaldehyde (Solarbio, Beijing, China) for 24 h, dehydrated, and embedded in paraffin. Sections (5 µm) were cut through the pupil and optic nerve axis and stained with hematoxylin and eosin (H&E). Retinal histopathological changes were graded on a scale of 0–4, as described previously [27].

### β-Galactosidase staining

Wild-type (WT) and EAU mice were killed and the spleen, liver, kidney, and eye were quickly dissected, and frozen in optimum cutting temperature compound (OCT) in liquid nitrogen, and stored at − 80 °C until sectioning. Sections (15 μm) were mounted on superfrost plus slides, fixed for 10 min in 2% paraformaldehyde/0.5% glutaraldehyde solution in phosphate buffered saline (PBS), briefly washed in PBS, and incubated in phosphate buffer (pH 6.0) containing potassium ferrocyanure, NaCl, MgCl_2_, and X-gal for 24 h at 37 °C. After color development, sections were dehydrated, coverslipped in permount, and imaged under a microscope.

### Western blot

For total protein extraction from spleen and retinal tissue, RIPA lysis buffer (Sigma-Aldrich, Missouri, USA) with protease mixture (Sigma-Aldrich, Missouri, USA) was used to obtain whole-cell lysates. Equal amounts of protein (35 µg) were separated using sodium dodecyl-sulfate polyacrylamide gel electrophoresis (SDS-PAGE). Proteins were transferred onto polyvinylidene fluoride (PVDF) membranes for Western blot analysis using rabbit anti-β-actin, rabbit anti-β-catenin, and rabbit anti-active catenin (Cell Signaling Technology). Chemiluminescent horseradish peroxidase substrate (Millipore, MA, USA) was used to detect antibody–antigen complexes.

### Quantitative real-time polymerase chain reaction (qPCR)

Total RNA was isolated from spleen, retina, and DCs using TRIzol reagent, according to the manufacturer’s instructions (Invitrogen, USA). Reverse transcription to cDNA was performed using RevertAid First Strand cDNA Synthesis reagents (Thermo Scientific, USA). All gene transcripts were analyzed using the SYBR green mix (Newbio industry, China) and detected using an ABI 7500 fast instrument (Applied Biosystems, CA). The relative mRNA expression in each sample was displayed as 2^−ΔΔCt^ values and was representative of at least three independent replicates. The following primer sequences were used for the real-time PCR analysis:

Lef-1 Forward: TCACTGTCAGGCGACACTTC

Reverse: ATGAGGTCTTTTGGGCTCCT

Tcf-1 Forward: GCTGCCATCAACCAGATCCT

Reverse: TGCATTTCTTTTTCCTCCTGTGG

Sox9 Forward: GACGTGCAAGCTGGGAAAGT

Reverse: CGGCAGGTATTGGTCAAACTC

Axin2 Forward: ATGAGTAGCGCCGTGTTAGTG

Reverse: GGGCATAGGTTTGGTGGACT

Wnt1 Forward: TGGCTGGGTTTCTGCTACG

Reverse: CCCGGATT TTGGCGTAT

Wnt3a Forward: ATACACCACCCAACCTCACG

Reverse: AGACACCATCCCACCAAACT

Wnt5a Forward: CAACTGGCAGGACTTTCTCAA

Reverse: CCTTCTCCAATGTACTGCATGTG

Wnt5b Forward: AAGTGTCATGGCGTCTCAG

Reverse: GGTTCCAACAGAGGGTTTTT

Kallistatin Forward: TAGGGCGGATCCGGTACCGAGGAGATCTGC

Reverse: TAGGGCCGGCCGCTATGGTTTCGT

DKK1 Forward: CAGCTCAATCCCAAGGATGT

Reverse: CAGGGGAGTTCCATCAAGAA

IL-6 Forward: AGCCAGAGTCCTTCAGAGAG

Reverse: GATGGTCTTGGTCCTTAGCC

IL-1βForward: TAAAGACCTCTATGCCAACACAGT

Reverse: CTGACTTGGCAGAGGACAAAG

IL-12 Forward: GAGCACTCCCCATTCCTACT

Reverse: GCATTGGACTTCGGTAGATG

IL-23 Forward: GGAAGCACGGCAGCAGAATA

Reverse: AACTTGAGGGAGAAGTAGGAATGG

TGF-β1 Forward: GTGTGGAGCAACATGTGGAACTCTA

Reverse: TTGGTTCAGCCACTGCCGTA

IL-10 Forward: CGGGAAGACAATAACTGCACCC

Reverse: CGGTTAGCAGTATGTTGTCCAGC

GAPDH Forward: ACCACAGTCCATGCCATCAC

Reverse: TCCACCACCCTGTTGCTGTA

### Splenic CD11c^+^ DC isolation

CD11c^+^ DCs were sorted from the spleens of WT mice or mice with EAU on day 3 using anti-mouse CD11c magnetic microbeads (Miltenyi Biotec, Auburn, CA) according to the manufacturer’s instructions. The purity of CD11c^+^ DCs was approximately 95%, and was determined by flow cytometry analysis.

### Splenic CD11c^+^ DC culture

CD11c^+^ DCs sorted from the spleens of mice with EAU on day 3 were cultured (10^6^ cells/ml) in Dulbecco’s modified Eagle’s medium for 24 h, and then stimulated with adenosine triphosphate (ATP) for 60 min. mRNA and culture supernatant were collected and subjected to qPCR and enzyme-linked immunosorbent assay (ELISA).

### IRBP, *M. tuberculosis*, and PTX stimulation CD11c^+^ DCs

CD11c^+^ DCs sorted from the spleens of WT mice were cultured (10^6^ cells/ml) with IRBP (20 µg/ml), *M. tuberculosis* (25 µg/ml), and PTX (5 ng/ml) for 24 h. The culture supernatants were collected for cytokine analysis by ELISA.

### ELISA

To measure cytokine concentrations in cell culture supernatants, the following ELISA kits purchased from R&D Systems (Minneapolis, MI, USA) were used: IL-1β, IL-12, and IL-10. The following ELISA kits were used to measure protein concentrations in mouse plasma: kallistatin (MyBioSource) and DKK1 (R&D Systems). ELISA assays were performed according to the manufacturer’s instructions.

### Naïve OT-II T cell isolation

CD4^+^ CD44^low^ CD62L^high^ CD25^−^ naïve OT-II T cells were purified by fluorescence activated cell sorting (FACS; FACSAria, BD Biosciences). CD4^+^ cells were then isolated using MACS beads (Dynabeads™ Untouched™ Mouse CD4 cells, Invitrogen, USA), according to the manufacturer’s instructions.

### Coculture of naïve OT-II CD4^+^ T cells with DCs

CD11c^+^ DCs (10^6^ cells/ml) sorted from WT mice were stimulated with PBS or PTX (25 μg/ml) for 8 h and washed with RPMI 1640 medium. For T cell proliferation assays, activated DCs (2 × 10^4^) were cultured with carboxyfluorescein diacetate, succinimidyl ester (CFSE)-labeled naïve OT-II CD4^+^ T cells at a ratio of 1:2 or 1:5, in the presence of ovalbumin 323–339 (OVA 323–339) peptide (2 μg/ml). After 4 days, cells were subjected to FACS. For T cell differentiation assays, activated DCs (2 × 10^4^) were cultured with naïve OT-II CD4^+^ T cells at a ratio of 1:2 in the presence of OVA peptide (2 μg/ml) and TGF-β (1 ng/ml; R&D). After 5 days, cells were collected and re-stimulated for intracellular cytokine staining followed by FACS analysis.

### T helper cell proliferation and differentiation

Forty-eight-well plates were pre-coated with anti-CD3 mAb (5 μg/ml; BioLegend, CA, USA) and anti-CD28 mAb (2 μg/ml; BioLegend, CA, USA) and incubated at 4 °C overnight. For T cell proliferation assays, 2.5 × 10^5^ CFSE-labeled CD4^+^ T cells were seeded in 48-well plates and cultured for 4 days. For Th17 cell differentiation assays, 2.5 × 10^5^ CD4^+^ T cells were seeded in 48-well plates and cultured in Th17-polarizing conditioned medium supplemented with IL-6 (20 ng/ml), TGF-β1 (1 ng/ml), anti-IL4 (10 μg/ml), and anti-IFN-γ (10 μg/ml) for 4 days. For Th1 cell differentiation assays, 2.5 × 10^5^ CD4^+^ T cells were seeded in 48-well plates and cultured in Th1-polarizing conditioned medium supplemented with IL-12 (20 ng/ml) and anti-IL4 (10 μg/ml) for 4 days.

### Isolation of infiltrating cells from the eyes

The eyes of mice with EAU were collected on day 21 post-immunization. After removal of the cornea, lens, optic nerve, and excess connective tissue, the remaining eye tissue was ground, followed by digestion with RPMI1640 containing 1 mg/ml collagenase D (Roche) for 1 h at 37 °C on a shaker at 220 rpm. A single cell suspension of inflamed eye tissue was obtained by passing the sample through a 70-µm strainer.

### Flow cytometry

Isolated splenocytes, ocular lymphocytes, human peripheral blood mononuclear cells (PBMCs), and T cells were resuspended in PBS containing 2% fetal bovine serum (FBS). Cells were either immediately analyzed or stimulated with 50 ng/ml phorbol 12-myristate 13-acetate (PMA), 500 ng/ml ionomycin, and 1 μg/ml brefeldin A for 5 h. For surface marker staining, cells were incubated with antibodies against CD11C, CD3, B220, MHC-II, CD80, CD86, and CD4 (BioLegend, CA, USA). For intracellular staining, cells were washed, fixed, permeabilized, and stained with active β-catenin, β-galactosidase, IFN-γ, IL-17a, and Foxp3, according to the manufacturer’s instructions. The appropriate isotype-matched mAbs were used as negative controls. Cells were detected using a FACS flow cytometer (BD Biosciences) and analyzed using flowjo_v10 software (FlowJo LLC, USA).

### Statistics

GraphPad Prism 9.0 (GraphPad Software Inc., San Diego, CA, USA) was used for statistical analyses. Multiple-group comparisons were performed using one-way or two-way analysis of variance (ANOVA) tests followed by Bonferroni’s post hoc test. Unpaired two-tailed Student’s *t* tests were used for comparisons of two conditions. Mann–Whitney *U* tests were used for comparing EAU clinical scoring. Data are expressed as mean ± standard deviation (SD). *P* values < 0.05 were considered significant.

## Results

### Wnt/β-catenin signaling is suppressed in EAU mice and uveitis patients

**Fig. 1:**
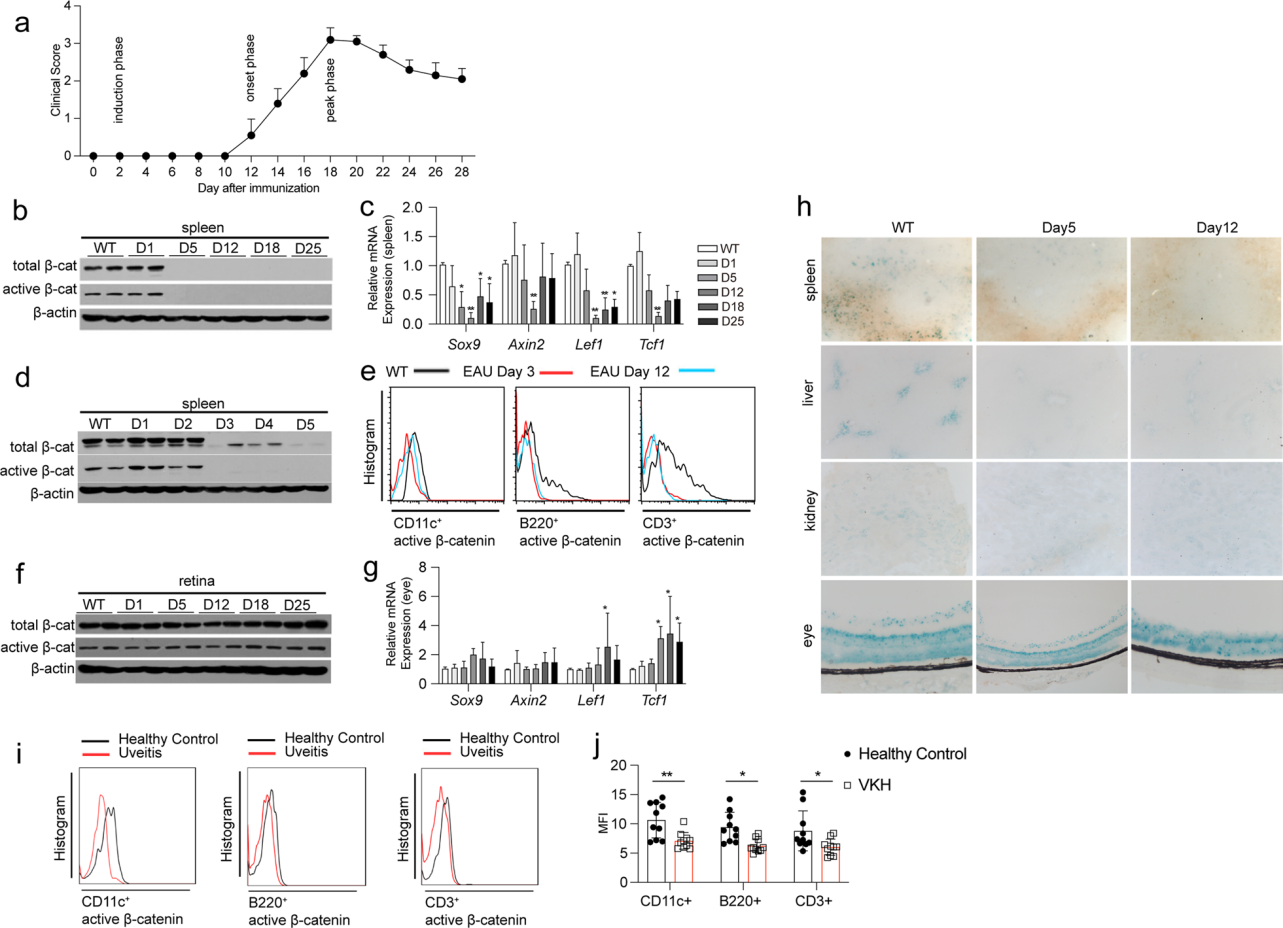
Wnt/β-catenin pathway activity in autoimmune uveitis. **a** Schematic of the clinical evaluation of EAU models (*n* = 10). **b** Detection of active β-catenin and total β-catenin levels in spleens from WT mice and mice with EAU on D1, D5, D12, D18, and D25 post-immunization using Western blotting (*n* = 6). **c** Expression of Wnt target genes, *Lef1*, *Sox9*, *Axin2*, and *Tcf1*, in spleens from WT mice and mice with EAU, at D1, D5, D12, D18, and D25 post-immunization, quantified using qPCR (*n* = 5). **d** Active β-catenin and total β-catenin levels in the spleens from WT mice and mice with EAU, at D1, D2, D3, D4, and D5 post-immunization, detected using Western blotting (*n* = 6). **e** Expression of active β-catenin in CD11c^+^ DCs, B220^+^ B cells, and CD3^+^ T cells in the spleens of WT mice and mice with EAU at D3 and D12 post-immunization, detected using FACS (*n* = 3). **f** Active β-catenin and total β-catenin levels in the retinas from WT mice and mice with EAU at D1, D5, D12, D18, and D25 post-immunization, detected using Western blotting (*n* = 5). **g** Expression of Wnt target genes, *Lef1*, *Sox9*, *Axin2*, and Tcf1, in retinas from WT mice and mice with EAU at D1, D5, D12, D18, and D25 post-immunization, quantified using qPCR (*n* = 5). **h** β-Galactosidase reporter gene expression is illustrated in blue color in the spleen, liver, kidney, and eyes of Wnt reporter mice (Axin2-LacZ) post-immunization on D3 and D12 detected using β-galactosidase staining (*n* = 3). **i** Expression of active β-cateninin in CD11c^+^ DCs, CD3^+^ T cells, and B220^+^ B cells from the PBMCs of acute VKH patients and healthy controls detected using FACS. **j** Mean fluorescence intensity (MFI) was measured from VKH patients (*n* = 10) and healthy controls (*n* = 10). Data are presented as mean ± SD. NS > 0.05, *p < 0.05, **p < 0.01

To determine the activation status of Wnt/β-catenin signaling in uveitis, EAU was induced in mice and the expression of β-catenin and Wnt target genes (*Lef1*, *Sox9*, *Axin2*, and *Tcf1*) measured at different stages of EAU, including the induction phase (days 1 and 5 post-immunization; D1 and D5), onset phase (day 12 post-immunization; D12), and peak phase (days 18 and 25 post-immunization; D18 and D25) (Fig. 1a). From D5, the levels of both active β-catenin and total β-catenin in the spleen decreased significantly (Fig. 1b). *Sox9* mRNA expression declined from D5, and *Tcf1* and *Lef1* expression decreased from D12 (Fig. 1c). To identify the exact timing of β-catenin downregulation, we measured active β-catenin and total β-catenin levels daily from days 1 to 5 post-immunization, and observed that both active and total β-catenin expression levels were suppressed significantly, from D3, in the spleen (Fig. 1d). Flow cytometry analysis revealed that active β-catenin levels in CD11c^+^ DCs, CD3^+^ T cells, and B220^+^ B cells were all downregulated at D3 and D12 in the spleen (Fig. 1e); however, both active and total β-catenin levels were unaltered in the eyes of EAU mice (Fig. 1f). Among the four Wnt signaling target genes assayed, the mRNA levels of *Tcf1* increased on D12, and Lef1 increased on D18 in the eyes (Fig. 1g). We also induced EAU in Wnt reporter mice (Axin2-LacZ) and found that β-gal expression decreased at D5 and D12 in the spleen, kidney, and liver (Fig. 1h). In Wnt report mice, β-gal expression in the eyes of EAU mice and WT mice was similar at D3 and D12 (Fig. 1h).

To explore the activation status of Wnt/β-catenin signaling in patients with uveitis, we collected PBMCs from healthy controls and patients with acute Vogt–Koyanagi–Harada disease (VKH) and assessed Wnt/β-catenin pathway activation in total PBMCs, CD11c^+^ DCs, CD3^+^ T cells, and B220^+^ B cells. We found that the levels of active β-catenin in the cells were reduced significantly in patients with VKH (Fig. 1i, j).

Overall, the results indicate that Wnt/β-catenin signaling is inhibited in DCs, T cells, and B cells at very early stages of EAU and in patients with acute uveitis.

### PTX inhibits Wnt/β-catenin signaling

**Fig. 2:**
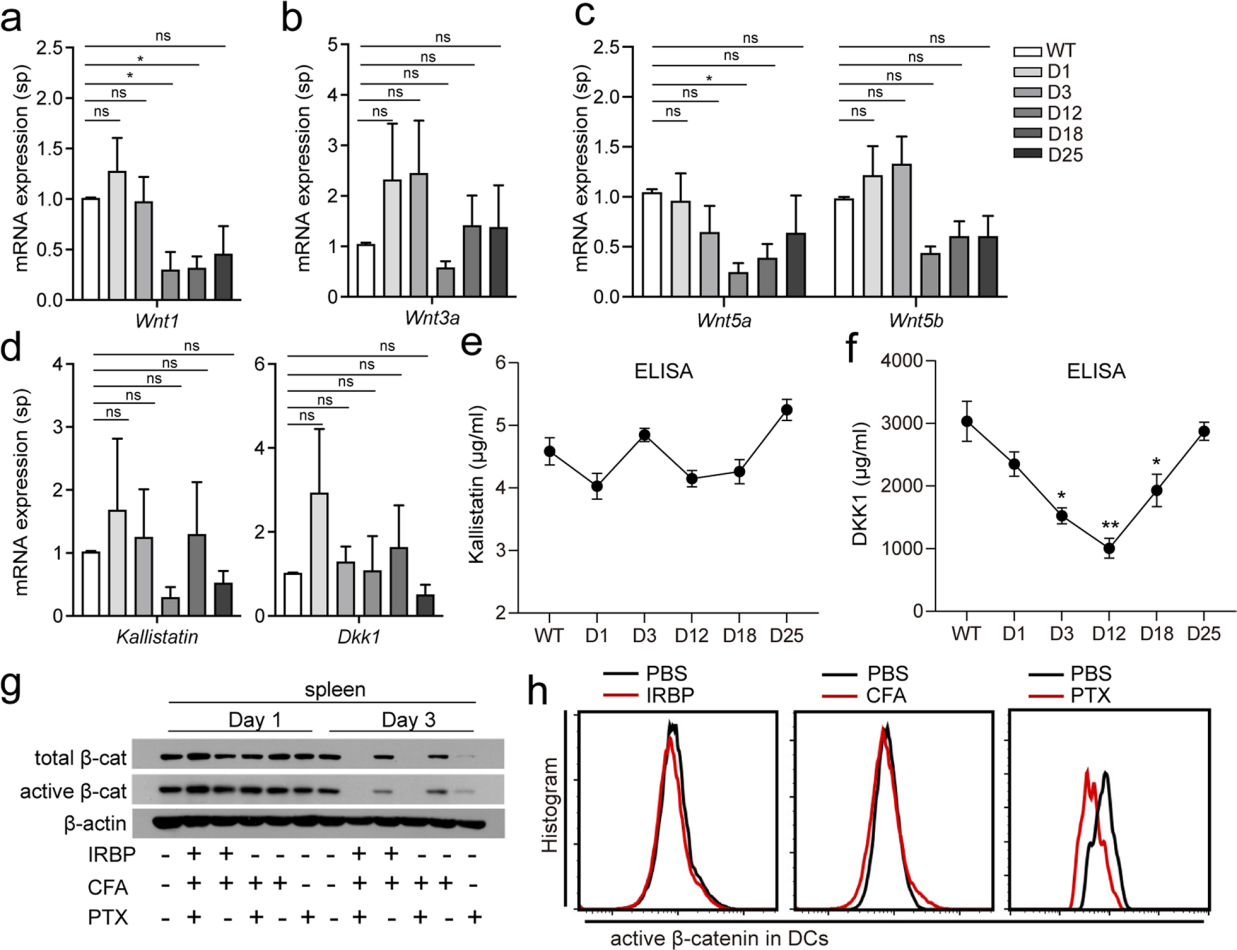
Changes in Wnt ligands and antagonists in mice with EAU after immunization and the effect of different elements used for immunization on β-catenin expression. **a**–**d** Expression of Wnt ligands (*Wnt1*, *Wnt3a*, *Wnt5a*, and *Wnt5b*) and Wnt antagonists (*kallistatin* and *DKK1*) in spleens from WT mice and mice with EAU on D1, D5, D12, D18, and D25 post-immunization quantified using qPCR (*n* = 5). **e**, **f** The levels of Kallistatin and DKK1 in plasma from WT mice and mice with EAU on D1, D5, D12, D18, and D25 post-immunization measured using ELISA (*n* = 7). **g** Expression of active β-catenin and total β-catenin in the spleens of WT mice immunized with CFA, PTX, and IRBP, separately or in combination, from D1 to D3 detected using Western blotting. **h** Expression of active β-catenin in CD11c^+^ DCs from the spleens of WT mice immunized with CFA, PTX, or IRBP on D3 post-immunization detected using FACS (*n* = 3). Data are presented as mean ± SD. NS > 0.05, *p < 0.05, **p < 0.01

To identify the factors that led to the downregulation of Wnt/β-catenin signaling, we assessed the expression of Wnt ligands (*Wnt1*, *Wnt3a*, *Wnt5a*, and *Wnt5b*) and Wnt antagonists (*kallistatin* and *DKK1*) in spleen and plasma. The results revealed that only the expression of *Wnt1* and *Wnt5a* decreased at D12 at the mRNA level, while the expression levels of *Wnt3a* and *Wnt5b* exhibited decreasing trends, from D12 to D25 (Fig. 2a–c). There were no significant differences in the mRNA levels of *Kallistatin* and *Dkk1* in the spleen (Fig. 2d). No significant changes in kallistatin levels in EAU mouse plasma were observed at any stages of the disease (Fig. 2e), whereas DKK1 levels decreased significantly from D3 to D18, when compared with those in WT mouse plasma (Fig. 2f). The results suggest that the changes in Wnt ligands and Wnt antagonists may not be associated with the inhibition of Wnt/β-catenin pathway that occurred in the early stage of EAU.

We also assessed the effects of different components used for immunization on Wnt/β-catenin pathway activity. We used CFA, PTX, and IRBP651-670 (IRBP), separately or in combination, to immunize mice. Immunization with PTX alone, PTX + CFA, or PTX + IRBP, suppressed β-catenin expression at D3 in the spleen significantly (Fig. 2g). The mice injected with CFA, or IRBP alone, did not suppress active β-catenin in DCs (Fig. 2h). The results indicate that PTX leads to inactivation of Wnt/β-catenin signaling.

### PTX programs DCs to induce pro-inflammatory factor secretion and uveogenic Th1/17 cell development in vivo

**Fig. 3:**
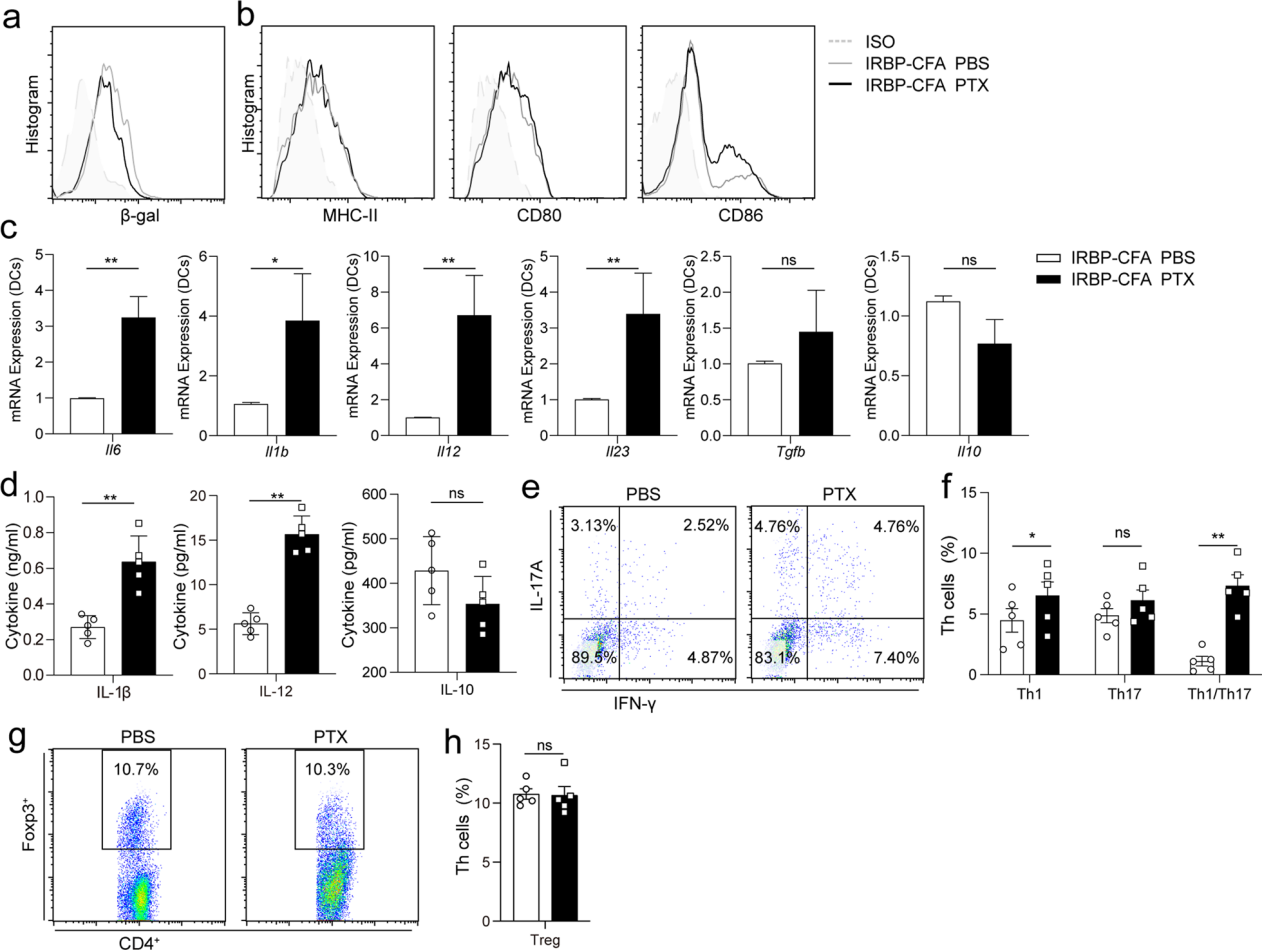
Effects of PTX treatment on DCs and T cells in vivo. **a** β-Galactosidase expression on D3 post-immunization in CD11c^+^DCs from the spleens of Axin2-reporter mice immunized with IRBP-CFA and injected intraperitoneally with PTX or PBS at day 0 and day 2 detected using FACS (*n* = 3). **b**–**d** WT mice were immunized with IRBP-CFA and injected intraperitoneally with PTX or PBS at day 0 and day 2. **b** Expression of MHC Class II, CD80, and CD86 in CD11c^+^ DCs sorted from the spleen on D3 post-immunization measured using FACS (*n* = 3). **c**, **d** Expression levels of inflammatory factors expressed in or secreted by CD11c^+^ DCs from the spleen at D3 post-immunization after 24 h of culture and 60 min of ATP stimulation detected using qPCR or ELISA (*n* = 5). **e**–**h** WT mice were immunized with IRBP-CFA and injected with PTX or PBS at day 0 and day 2. **e** Representative FACS plot showing the expression of IFN-γ and IL-17A in draining lymph node (DLN)-derived CD4^+^ T cells on D5 post-immunization (*n* = 5). **f** Percentages of the Th1 and Th17 cell subpopulations in DLN-CD4^+^ T cells (*n* = 5). **g** Representative FACS plot showing the expression of Foxp3 in DLN-derived CD4^+^ T cells on D5 post-immunization (*n* = 5). **h** Percentages of Treg cell subpopulations among DLN-CD4^+^ T cells (*n* = 5). The data shown are representative of three independent experiments. Data are presented as the mean ± SD. NS, *p* > 0.05, *p < 0.05, **p < 0.01

The secretion of pro-inflammatory factors by DCs is a key step in initiating immune response [28]. To explore the effect of PTX-induced Wnt/β-catenin inhibition on the response of DCs in vivo, we injected mice with or without PTX at D0 and D2. The inhibition of Wnt/β-catenin signaling by PTX was confirmed using DCs from Wnt reporter mice at D3 (Fig. 3a). At D3, we sorted CD11c^+^ DCs from the spleen and examined the spontaneous production of pro- or anti-inflammatory factors. Flow cytometric analysis revealed that PTX inhibited the expression of CD86 significantly, but not MHC-II or CD80 (Fig. 3b). *Il1β*, *Il6*, *Il12*, and *Il23* mRNA levels in DCs were upregulated following PTX injection, while no significant changes were observed for *Tgfb* or *Il10* expression (Fig. 3c). By performing an ELISA for the culture supernatant, we confirmed that PTX prompted the secretion of IL-1β and IL-12, but not IL-10 (Fig. 3d), demonstrating that PTX induces the secretion of inflammatory factors from DCs.

DCs determine the fate of T cells by producing various inflammatory cytokines [29]; therefore, we explored the effects of PTX on the development of T helper cells in vivo. We detected Th1, Th17, and Treg cells in the spleen at D5 in PTX- or PBS-treated groups and found that PTX injection increased the proportions of Th1 and Th1/Th17 cells significantly (Fig. 3e, f) without affecting the proportions of Th 17 or Treg cells (Fig. 3g, h). Overall, the data demonstrate that PTX induces the development of pathogenic Th1 cells in vivo.

### PTX programs DCs to induce pro-inflammatory factor secretion and Th1/17 cell proliferation and differentiation in vitro

**Fig. 4:**
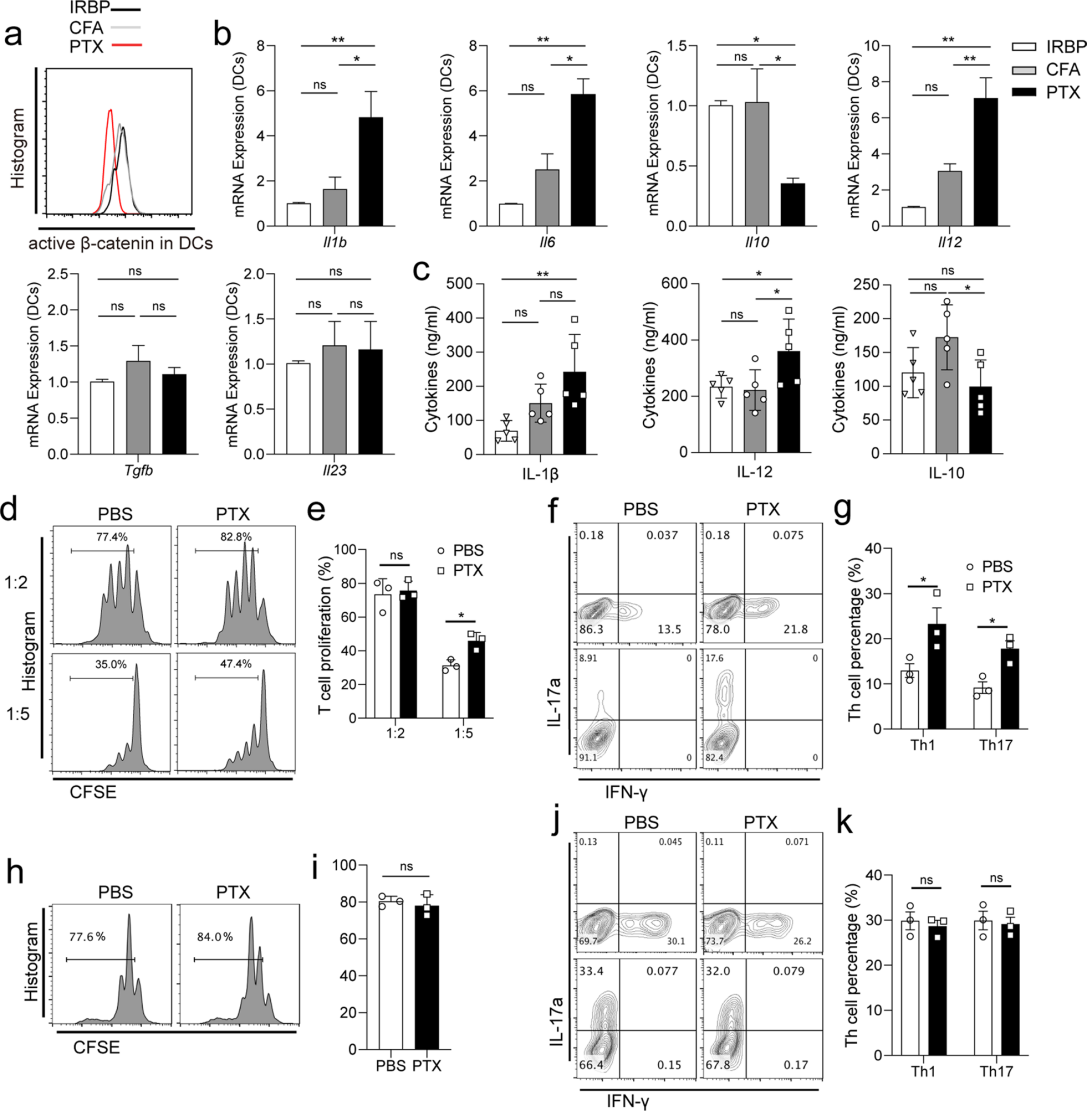
Effects of PTX treatment on DCs and T cells in vitro. **a**–**c** CD11c^+^ DCs were isolated from WT mice and stimulated with IRBP (20 µg/ml), CFA (25 µg/ml), and PTX (5 ng/ml) for 24 h in culture medium. **a** Expression levels of active β-catenin detected using FACS (*n* = 3). **b**, **c** Expression levels of inflammatory factors expressed in or secreted by CD11c^+^ DCs detected using qPCR or ELISA (*n* = 5). **d**, **e** CD11c^+^ DCs from the spleens of WT mice were stimulated with PBS or PTX for 8 h and then co-cultured with CFSE-labeled naïve OT-II T cells at 1:2 or 1:5 ratios for 4 days in the presence of OVA peptide. **d** Representative FACS plot showing CFSE-labeled naïve OT-II T cell proliferation stimulated by PBS- or PTX-treated DCs. **e** The percentages of CFSE-labeled proliferating T cells (*n* = 3). **f**, **g** CD11c^+^ DCs from the spleens of WT mice were stimulated with PBS or PTX for 24 h and then co-cultured with naïve OT-II T cells in a 1:2 ratio in the presence of OVA peptide for 5 days. **f** Representative FACS plot showing IFN-γ^+^ and IL-17A^+^ CD4^+^ T cell differentiation stimulated by PBS- or PTX-treated DCs. **g** The percentages of Th17 and Th1 differentiated cells (*n* = 3). **h**, **i** CFSE-labeled T cells were stimulated with CD3/28 and treated with PBS or PTX for 96 h. **h** Representative FACS plot showing CFSE-labeled naïve OT-II T cell proliferation following treatmeant with PBS or PTX. **i** The percentages of CFSE-labeled proliferating T cells (*n* = 3). **j**, **k** CFSE-labeled T cells were cultured under Th1 or Th17 differentiation conditions and treated with PBS or PTX for 96 h. **j** Representative FACS plot showing naïve OT-II T cell differentiation following treatment with PBS or PTX. **k** The percentages of differentiated T cells (*n* = 3). Data shown are representative of thre independent experiments. Data are mean ± SD. NS, *p* > 0.05, *p < 0.05, **p < 0.01

We further investigated the effect of PTX on DC function in vitro. We purified splenic DCs and pre-treated them with IRBP, *M. tuberculosis* (active ingredient of CFA), and PTX for 24 h. We then extracted mRNA from the cultured cells for qPCR analysis and collected the culture supernatant for ELISA. As shown in Fig. 4a, PTX inhibited the expression of active β-catenin in DCs in vitro significantly*.* Compared with the IRBP651-670 and TB treatments, PTX treatment enhanced *Il1β*, *Il6*, and *Il12* mRNA expression, but inhibited *Il10* mRNA expression*,* in DCs (Fig. 4b). Compared to IRBP651-670 treatment, TB treatment enhanced *Il12* mRNA expression (Fig. 4b). Neither TB nor PTX treatment affected the expression of *TGF-β* or *Il23* (Fig. 4b). We further used ELISA to confirm that PTX treatment promoted IL-1β and IL-12 secretion, but inhibited IL-10 secretion, from DCs (Fig. 4c). The data indicate that PTX is essential for DCs to produce pro-inflammatory cytokines and inhibit anti-inflammatory cytokines. We then investigated whether PTX affected T cell biology by mediating the responses of DCs in vitro. We began by assessing the effect of PTX-treated DCs on naïve OT-II CD4^+^ T cell proliferation. When PTX-pretreated DCs were co-cultured with CFSE-labeled T cells at a 1:5 ratio, T cell proliferation was enhanced significantly when compared with PBS-pretreated DCs (Fig. 4d, e). Subsequently, we evaluated the effect of PTX-treated DCs on the differentiation of naïve OT-II CD4^+^ T cells. When DCs were co-cultured with T cells at a 1:2 ratio, T cell differentiation toward both Th1 and Th17 lineages was promoted by PTX treatment significantly (Fig. 4f, g). Conversely, PTX treatment did not directly inhibit CD3/28-induced T cell proliferation (Fig. 4h, i) or differentiation (Fig. 4j, k). Overall, the data suggest that PTX treatment promotes T cell proliferation and differentiation by regulating DC response.

### β-Catenin deletion in DCs enhances the severity of EAU

**Fig. 5:**
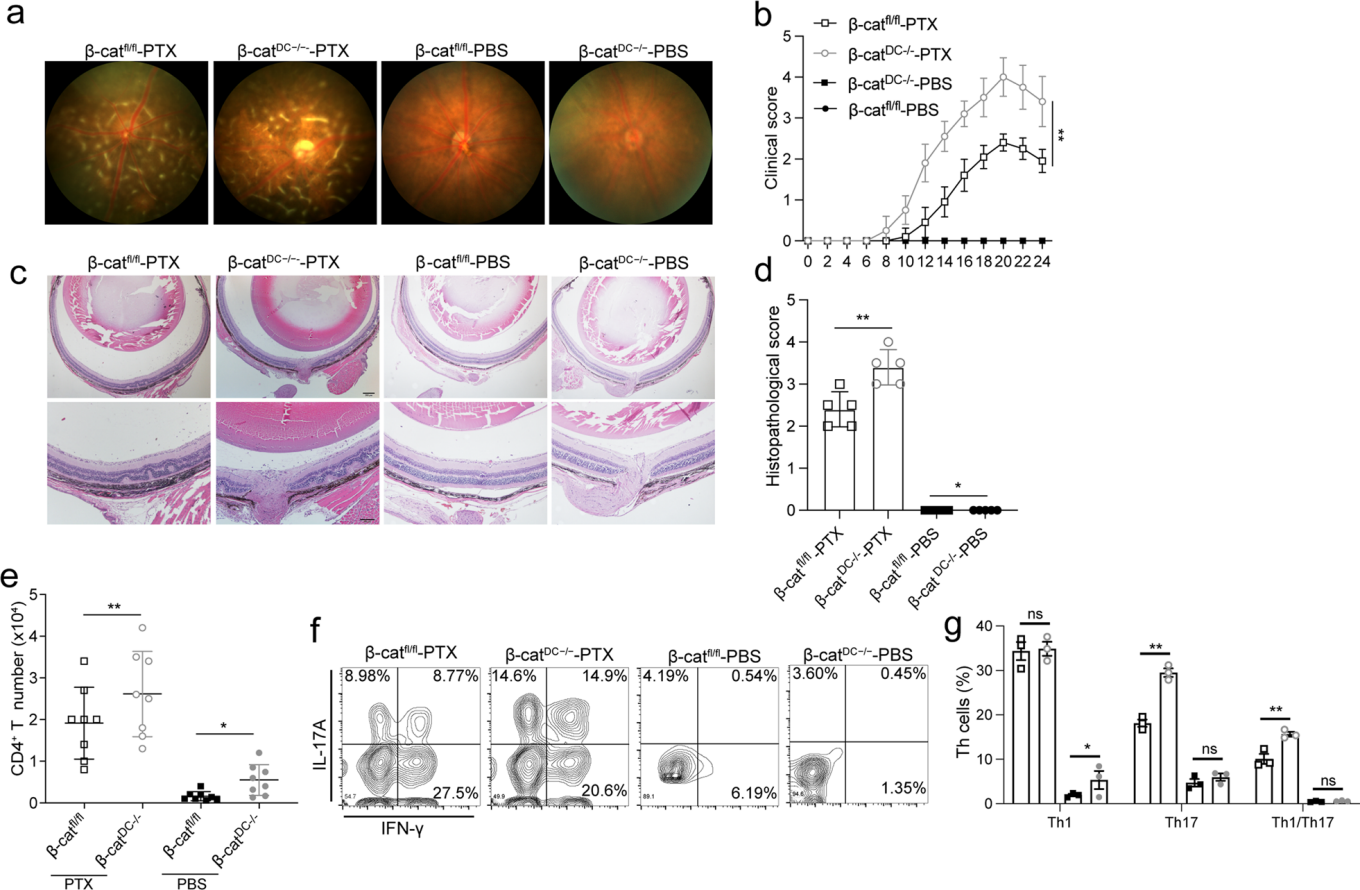
Effects of conditional β-catenin knockout in DCs on EAU pathogenesis. β-cat^fl/fl^ and β-cat^DC−/−^ mice were immunized with IRBP-CFA and intraperitoneally injected with PTX or PBS on day 0 and day 2. **a**–**c** The fundus digital image, clinical scores (*n* = 10), and histological staining (*n* = 5) of β-cat^fl/fl^ IRBP-CFA-PBS-treated mice, β-cat^DC−/−^ IRBP-CFA-PBS-treated mice, β-cat^fl/fl^ IRBP-CFA-PTX-treated mice, and β-cat^DC−/−^ IRBP-CFA-PTX-treated mice. **d** Histopathological scores for EAU in β-cat^fl/fl^ IRBP-CFA-PBS-treated mice, β-cat^DC−/−^ IRBP-CFA-PBS-treated mice, β-cat^fl/fl^ IRBP-CFA-PTX-treated mice, and β-cat^DC−/−^ IRBP-CFA-PTX-treated mice at day 21 post-immunization (*n* = 5). **e** Total number of CD4^+^ T lymphocytes infiltrating the eye on day 21 post-immunization (*n* = 8). **f** Expression of IFN-γ and IL-17A in eye-derived CD4^+^ T cells from β-cat^fl/fl^ PBS-treated mice, β-cat^DC−/−^ PBS-treated mice, β-cat^fl/fl^ PTX-treated mice, and β-cat^DC−/−^ PTX-treated mice detected using FACS at D21 post-immunization. **g** The percentages of IL17^+^ cells and IFN-γ^+^ cells in eye-derived CD4^+^ T cells from β-cat^fl/fl^ PBS-treated mice, β-cat^DC−/−^ PBS-treated mice, β-cat^fl/fl^ PTX-treated mice, and β-cat^DC−/−^ PTX-treated mice (*n* = 3). Data are representative of three independent experiments. Data are mean ± SD. NS, *p* > 0.05, *p < 0.05, **p < 0.01

As described above, PTX treatment may mediate the initiation of autoimmune inflammation by inhibiting Wnt/β-catenin signaling in DCs. We next explored the effect of deleting β-catenin in DCs on EAU pathogenesis. We generated a DC-specific deletion of β-catenin (β-cat^DC−/−^) by crossing β-catenin floxed mice (β-cat^fl/fl^) with CD11c-cre mice. β-cat^fl/fl^ and β-cat^DC−/−^ mice were immunized with IRBP-CFA and injected with PTX or PBS on day 0 and day 2. The fundus digital image and clinical score results revealed that IRBP-CFA-PTX-injected β-cat^DC−/−^ mice developed more severe uveitis than IRBP-CFA-PTX-injected β-cat^fl/fl^ mice (Fig. 5a, b). Histological staining results showed that IRBP-CFA-PTX-injected β-cat^DC−/−^ mice developed more severe uveitis than IRBP-CFA-PTX-injected β-cat^fl/fl^ mice (Fig. 5c, d). Flow cytometry analysis revealed a greater accumulation of infiltrating CD4^+^ T cells in the eyes of IRBP-CFA-PTX-injected β-cat^DC−/−^ mice than in IRBP-CFA-PTX-injected β-cat^fl/f^ mice (Fig. 5e). The proportions of Th17, Th1, and Th1/Th17 (IFN-γ and IL-17A double-positive) cells were also significantly higher in the eyes of IRBP-CFA-PTX-injected β-cat^DC−/−^ mice than in the eyes of IRBP-CFA-PTX-injected β-cat^fl/fl^ mice (Fig. 5f, g). However, the injection of IRBP-CFA-PBS into β-cat^DC−/−^ or β-cat^fl/fl^ mice did not induce any clinical symptoms in the animals, indicating that PTX promotes EAU development via multiple pathways. Nonetheless, the data demonstrate that blocking the Wnt/β-catenin pathway in DCs enhances EAU pathologies.

### The GSK3 inhibitor, BIO, rescues PTX-induced Wnt/β-catenin inhibition

**Fig. 6:**
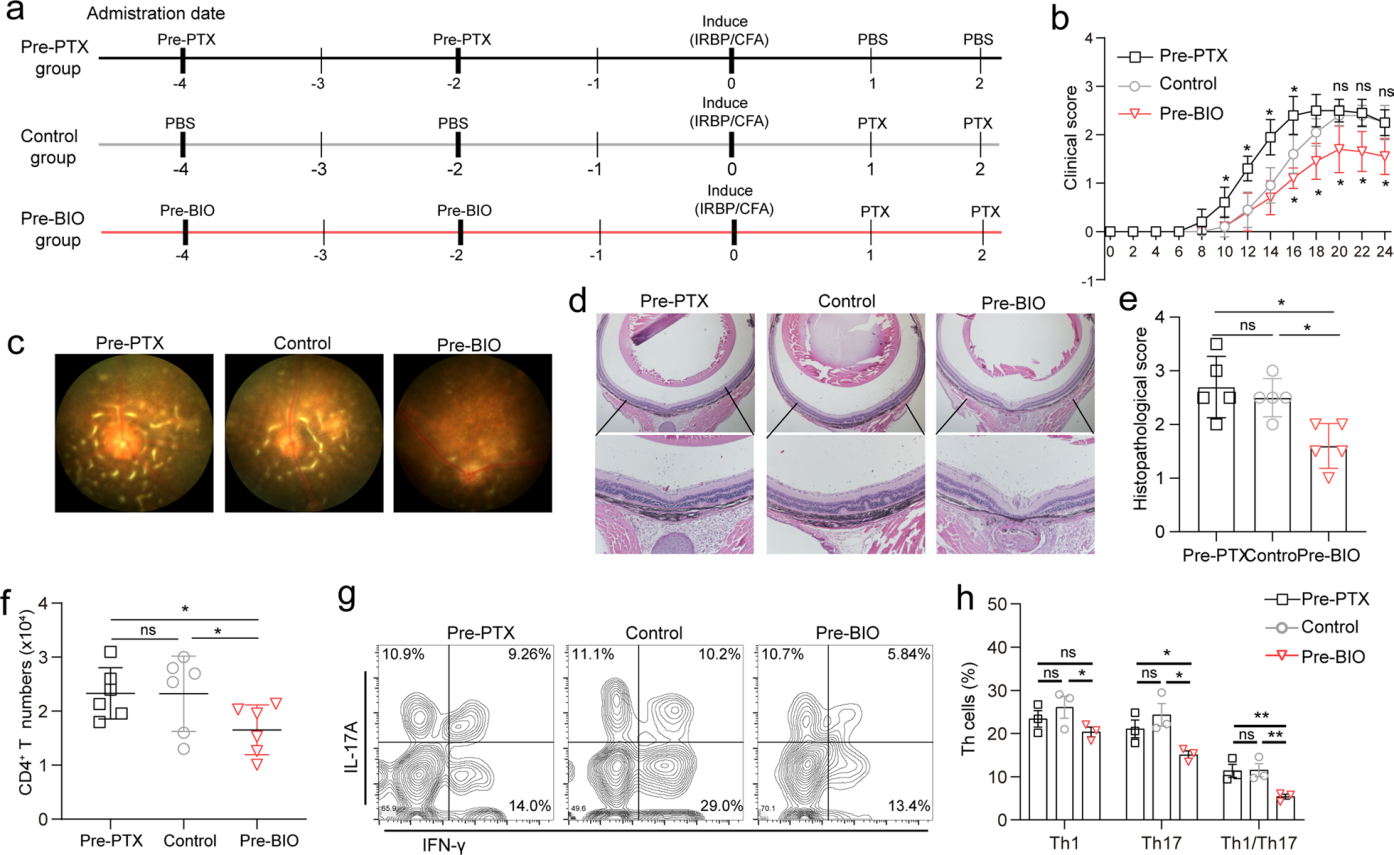
Effect of PTX pretreatment and BIO treatment on EAU development. **a** Diagram of the time of EAU induction and PTX and BIO administration. **b** EAU clinical scores of PTX-pre-treated mice, control mice, and BIO-pretreated mice recorded daily from day 6 to day 25 post-immunization (*n* = 6). **c** Representative fundus image of PTX-pre-treated mice, control mice, and BIO-pre-treated mice on day 21 after immunization. **d** Pathological representatives of hematoxylin and eosin (H&E) staining of the eye sections from PTX-pre-treated mice, control mice, and BIO-pre-treated mice on day 21 after immunization. **e** Histopathological scores for EAU in PTX-pre-treated mice, control mice, and BIO-pre-treated mice on day 21 post-immunization (*n* = 5). **f** Total number of CD4^+^ T lymphocytes infiltrating the eye on day 21 post-immunization (*n* = 6). **g** Expression of IFN-γ and IL-17A in eye-derived CD4^+^ T cells from PTX-pre-treated mice, control mice, and BIO-pre-treated mice detected using FACS on D21 post-immunization (*n* = 3). **h** The percentages of IL-17^+^ cells and IFN-γ^+^ cells in eye-derived CD4^+^T cells from PTX-pre-treated mice, control mice, and BIO-pre-treated mice (*n* = 3). Data are representative of three independent experiments. Data mean ± SD. NS > 0.05, *p < 0.05, **p < 0.01

6-Bromoindirubin-3′-oxime (BIO) is a GSK3 inhibitor widely used to mimic the activation of the Wnt signaling cascade [30]. We explored the effect of pre-administration of PTX or BIO on EAU pathology (Fig. 6a). Compared to the controls, mice pre-treated with PTX exhibited earlier disease onset, while pretreatment with BIO reduced inflammation severity (Fig. 6b). The fundus digital image and histological staining results showed that the fundus inflammation was milder in BIO-pretreated mice than in the control group (Fig. 6c–e). Flow cytometry analysis revealed that BIO pretreatment suppressed the infiltration of total CD4^+^ T cells, including Th1, Th17, and Th1/Th17 cells, in the eye (Fig. 6f–h). The results indicate that inhibiting the Wnt/β-catenin pathway in advance via PTX primes the mice for an autoimmune response and advances the onset of the disease. Conversely, mice treatment with a Wnt/β-catenin pathway activator limits the inhibitory effect of PTX on Wnt/β-catenin signaling, and ameliorates inflammation severity.

## Discussion

Wnt/β-catenin signaling plays a key role in the pathogenesis of autoimmune diseases by programing DCs; however, the factors regulating the Wnt/β-catenin pathway in DCs have remained elusive, and the role of Wnt/β-catenin signaling in autoimmune uveitis had never been reported. In the present study, PTX treatment suppressed Wnt/β-catenin signaling in DCs during the induction phase of EAU. Additionally, downregulation of the Wnt/β-catenin pathway induced the maturation of DCs and the secretion of inflammatory cytokines, which, in turn, stimulated the pathogenic Th1/Th17 cells and promoted an autoimmune response.

The function of DCs, proportions of T effector/T regulatory cells, and the expression of inflammatory factors are all closely associated with the occurrence of uveitis [31]. The EAU mouse model is an animal model commonly used for the investigation of autoimmune ocular diseases [32]. During the induction stage, the peripheral immune system becomes activated, DCs mature, and autoreactive T cells proliferate and differentiate [33]. During the effect phase, activated autoreactive T cells infiltrate the retina and interact with local parenchymal cells, microglia and astrocytes, which further recruit non-specific infiltrating cells, leading to an inflammatory cascade and retinal damage through the secretion of inflammatory cytokines and chemokines [34–36]. In the present study, the Wnt/β-catenin signaling was inhibited as early as 3 days after immunization (i.e., at the beginning of the induction phase) in the spleens of EAU mice. The level of β-catenin in PBMCs isolated from patients with acute uveitis was also reduced. Decreased β-catenin activity has previously been reported in leukocytes from lupus-prone mice and patients with systemic lupus erythematosus [37]. We also observed that decreased β-catenin activity inhibited the expression of Wnt-associated genes, such as *Sox9*, *Axin2*, *Tcf1*, and *Lef1*. Transcription factors TCF1 and LEF1 could regulate the proliferation, apoptosis, and function of DCs, Treg cells, and CD8^+^ T cells [38–40]. Sox9 regulated by Wnt/β-catenin plays a key role in the polarization of macrophages [41]. The results indicate that the downregulation of Wnt/β-catenin signaling before disease onset may be an event triggering autoimmune responses.

The effect of Wnt/β-catenin signaling on the immune system, including DCs, has been studied extensively. Recent studies have highlighted that Wnt/β-catenin signaling regulates the delicate immune balance by reprogramming DCs to a regulatory state [9]. Activation of Wnt/β-catenin signaling can condition DCs to produce high levels of regulatory factors, such as IL-10 and TGF-β, and low levels of inflammatory cytokines, including IL-6, IL-12, and TNF-α [42]. Such regulatory DCs can induce regulatory T cells and suppress the Th1/Th17 cell response, thereby protecting mice against autoimmune responses [10, 11, 43]. Conversely, mice that specifically lack Wnt/β-catenin signaling in DCs are more susceptible to antigen-induced autoimmune diseases. In both mouse models and patients with autoimmune hepatitis, Wnt/β-catenin signaling was deficient in hepatic DCs. Wnt ligand engagement restored the immunoregulatory phenotype of hepatic DCs by reactivating the Wnt/β-catenin pathway, thus alleviating the severity of autoimmune hepatitis [13]. Our results further support that downregulation of Wnt/β-catenin signaling in DCs plays a key role in inducing autoimmune responses. Although Wnt/β-catenin signaling was inhibited in the DCs, B cells, and T cells of mouse spleens in the present study, the downregulation of Wnt/β-catenin signaling did not directly affect the response of T cells in vitro, but promoted T cell proliferation and differentiation by inducing the maturation of DCs. Using DC-specific deletion β-catenin (β-cat^DC−/−^) mice, our in vivo experiments confirmed that β-catenin deletion in DCs enhanced EAU severity. One possible limitation of this study is that we only used CD11c as marker for DCs. Although CD11c is commonly used as a DC marker [44, 45], it is also expressed by other immune cells such as macrophage, B and T cells, and it is better to combine CD11c with other markers such as MHCII, CD11b, or CD103 to identify different subsets of DCs specifically [46, 47].

Previous work has demonstrated that PTX treatment can promote the maturation of DCs in vitro and upregulate their production of co-stimulatory molecules [48]; that way, PTX mediates the production of pro-inflammatory cytokines, such as IL-6, IL-12, and IL-1β, in DCs, and increases T cell responses, which may lead to autoimmune diseases, in vivo [49–52]. Alternatively, PTX could be selectively toxic to the endogenous tolerogenic cDC1 population, thereby removing the restraining effect of the DC subpopulation on immunogenicity [53]. Gαi/o proteins link to Frizzled-1, and the inhibition of Gαi/o proteins by PTX blocks Wnt-3A-induced Wnt/β-catenin signal activation [54, 55]. Therefore, the mechanism of β-catenin downregulation induced by PTX treatment may be attributed to the ADP-ribosyltransferase activity of PTX, which leads to the uncoupling of Frizzled-1 from the Gαi/o proteins and the subsequent inhibition of Wnt signaling.

Infections and human autoimmune diseases have multifaceted and multidirectional relationships [56]. Indeed, infection is believed to trigger autoimmune responses; for instance, cytomegalovirus (CMV) infection may promote the development of systemic lupus erythematosus (SLE) [57, 58]; CMV infection may stimulate the generation of T cells that cross-react with tyrosinase through a molecular mimicry mechanism, thereby inducing VKH pathogenesis [59]; activation of B cells by Epstein–Barr virus infection may be an early step in SLE pathogenesis [60–62]; Epstein–Barr viral infection may be involved in the pathogenesis of uveitis [63]; influenza viral infection is highly correlated with the occurrence of Guillain–Barré syndrome [64, 65] and bacterial infections, including *Campylobacter*, *Streptococcus pyogenes*, and *Staphylococcus aureus*, have been shown to be associated with the occurrence of multiple sclerosis, chronic rheumatic heart disease, Sjogren syndrome, VKH, and other autoimmune diseases [66–70]. The Wnt/β-catenin pathway is believed to be an important target of virulence factors produced by viruses, bacteria, and parasites [71]. The mechanisms by which such virulence factors interfere with Wnt/β-catenin activity are diverse and include the repression of Wnt inhibitors via epigenetic modification of histones [72], blocking of Wnt–Frizzled ligand binding [73], activation or inhibition of β-catenin nuclear translocation, and inhibition of Axin-1 expression (which promotes β-catenin activity) [74]. Notably, we observed that Wnt/β-catenin signaling was also inhibited in PBMCs isolated from patients with acute VKH. Collectively, although substantial evidence is insufficient, these data highlight a potential route via which infection can trigger apparent autoimmunity, and the results suggest that PTX may mimic the roles of virulence factors in humans to induce an autoimmune response, at least in part, by inhibiting Wnt/β-catenin signaling in DCs.

Su et al. reported that pretreatment of animals with PTX before adoptive transfer could protect from T cells from transferred EAU (tEAU), which is different from antigen-induced EAU (aEAU) [25]. They also reported that injection of PTX during the migration of effector T cells could block the induction of aEAU [25, 75]. The protective effect of PTX shown in tEAU and aEAU is associated with inhibition of lymphocyte migration. The data suggest that PTX may exert inhibitory or enhancing effects on autoimmune response PTX, with one or the other predominating at different stages. In the present study, PTX was injected before or at the same time as immunization. We believe that at this time PTX may mainly promote immune response by inhibiting the Wnt pathway. In an adoptive transfer EAU model, although no adjuvants are used for the induction of uveitis, the antigen specific T cells or DCs used for adoptive transfer EAU model are isolated from aEAU [76], in which the DCs have been pretreated by PTX, and T cells have been primed by PTX-treated DCs. Therefore, without adjuvants, such transferred antigen specific T cells or DCs already possess function similar to those of cells in aEAU in inducing uveitis.

After stimulation, DCs express co-stimulation molecules, such as CD80 and CD86 [77]. Both CD80 and CD86 facilitate the proliferation of T cells, and CD86 is associated with the Th17 response [78]. Dysregulation of CD80 and/or CD86 occurs in diverse autoimmune diseases, including EAE, RA, and EAU [79, 80]. Cytokines secreted by DCs, such as IL-12, IL-6, TGF-β, IL-10, IL-23, and IL-1β, may determine the fate of T cells [81]. IL-6 and TGF-β promote the polarization of Th17 cells [82], and IL-12 is a the key molecule for the formation of Th1 cells [83]. Conversely, IL-1β, TGF-β, and IL-23 play crucial roles in the development of pathogenic T cells [84–86]. There is also evidence that PTX may stimulate DCs to secrete IL-1β and regulate the formation of IL-17 and IFN-γ double-positive T cells [50]. In the present study, PTX induced DCs to express CD86, IL-6, IL-12, and IL-1β in vivo and in vitro. Simultaneously, PTX strongly induced the formation of IL-17 and IFN-γ double-positive T cells, which may be the result of increased IL-1β secretion by PTX stimulation.

## Conclusion

Overall, according to the results of the present study, during the induction phase of EAU, PTX inhibited Wnt/β-catenin signaling in DCs. The inhibition of the Wnt pathway promoted EAU pathogenesis by inducing the maturation of DCs and their secretion of inflammatory factors that promote uveitogenic T cells. Consequently, PTX-induced inhibition of Wnt/β-catenin signaling plays a key role in inducing autoimmune responses in EAU, providing new insights into the pathogenesis of autoimmune responses, as well as a novel immunotherapy target for autoimmune diseases.

## Data Availability

The datasets supporting the conclusions of this article are included within the article.

## References

[1] Zhong Z, Su G, Kijlstra A, Yang P (2021). Activation of the interleukin-23/interleukin-17 signalling pathway in autoinflammatory and autoimmune uveitis. Prog Retin Eye Res.

[2] Ke Y, Jiang G, Sun D, Kaplan HJ, Shao H (2011). Anti-CD3 antibody ameliorates experimental autoimmune uveitis by inducing both IL-10 and TGF-beta dependent regulatory T cells. Clin Immunol.

[3] Christofi M, Le Sommer S, Mölzer C, Klaska IP, Kuffova L, Forrester JV (2021). Low-dose 2-deoxy glucose stabilises tolerogenic dendritic cells and generates potent in vivo immunosuppressive effects. Cell Mol Life Sci.

[4] Heuss ND, Lehmann U, Norbury CC, McPherson SW, Gregerson DS (2012). Local activation of dendritic cells alters the pathogenesis of autoimmune disease in the retina. J Immunol.

[5] Sakoda Y, Nagai T, Murata S, Mizuno Y, Kurosawa H, Shoda H (2016). Pathogenic function of herpesvirus entry mediator in experimental autoimmune uveitis by induction of Th1- and Th17-type T cell responses. J Immunol.

[6] Sinclair C, Bommakanti G, Gardinassi L, Loebbermann J, Johnson MJ, Hakimpour P (2017). mTOR regulates metabolic adaptation of APCs in the lung and controls the outcome of allergic inflammation. Science.

[7] Roe MM, Hashimi M, Swain S, Woo KM, Bimczok D (2020). p38 MAPK signaling mediates retinoic acid-induced CD103 expression in human dendritic cells. Immunology.

[8] Wang H, Hu X, Huang M, Liu J, Gu Y, Ma L (2019). Mettl3-mediated mRNA m^6^A methylation promotes dendritic cell activation. Nat Commun.

[9] Manicassamy S, Reizis B, Ravindran R, Nakaya H, Salazar-Gonzalez RM, Wang YC (2010). Activation of beta-catenin in dendritic cells regulates immunity versus tolerance in the intestine. Science.

[10] Manoharan I, Hong Y, Suryawanshi A, Angus-Hill ML, Sun Z, Mellor AL (2014). TLR2-dependent activation of beta-catenin pathway in dendritic cells induces regulatory responses and attenuates autoimmune inflammation. J Immunol.

[11] Suryawanshi A, Manoharan I, Hong Y, Swafford D, Majumdar T, Taketo MM (2015). Canonical wnt signaling in dendritic cells regulates Th1/Th17 responses and suppresses autoimmune neuroinflammation. J Immunol.

[12] Wang J, Wang Y, Zhang H, Chang J, Lu M, Gao W (2020). Identification of a novel microRNA-141-3p/Forkhead box C1/beta-catenin axis associated with rheumatoid arthritis synovial fibroblast function in vivo and in vitro. Theranostics.

[13] Tan K, Xie X, Shi W, Miao L, Dong X, Yang W (2020). Deficiency of canonical Wnt/beta-catenin signalling in hepatic dendritic cells triggers autoimmune hepatitis. Liver Int.

[14] Tang S, Chen S, Huang B, Jiang J, Wen J, Deng Y (2019). Deoxynivalenol induces inhibition of cell proliferation via the Wnt/beta-catenin signaling pathway. Biochem Pharmacol.

[15] Draganova K, Zemke M, Zurkirchen L, Valenta T, Cantù C, Okoniewski M (2015). Wnt/beta-catenin signaling regulates sequential fate decisions of murine cortical precursor cells. Stem Cells.

[16] Kim JA, Kang YJ, Park G, Kim M, Park YO, Kim H (2009). Identification of a stroma-mediated Wnt/beta-catenin signal promoting self-renewal of hematopoietic stem cells in the stem cell niche. Stem Cells.

[17] Staal FJ, Luis TC, Tiemessen MM (2008). WNT signalling in the immune system: WNT is spreading its wings. Nat Rev Immunol.

[18] Swafford D, Manicassamy S (2015). Wnt signaling in dendritic cells: its role in regulation of immunity and tolerance. Discov Med.

[19] Reguart N, He B, Taron M, You L, Jablons DM, Rosell R (2005). The role of Wnt signaling in cancer and stem cells. Future Oncol.

[20] Daniels DL, Weis WI (2002). ICAT inhibits beta-catenin binding to Tcf/Lef-family transcription factors and the general coactivator p300 using independent structural modules. Mol Cell.

[21] Liu X, Zhang B, McBride JD, Zhou K, Lee K, Zhou Y (2013). Antiangiogenic and antineuroinflammatory effects of kallistatin through interactions with the canonical Wnt pathway. Diabetes.

[22] Holbourn KP, Shone CC, Acharya KR (2006). A family of killer toxins. Exploring the mechanism of ADP-ribosylating toxins. FEBS J.

[23] Iwanami K, Matsumoto I, Tanaka Y, Inoue A, Goto D, Ito S (2008). Arthritogenic T cell epitope in glucose-6-phosphate isomerase-induced arthritis. Arthritis Res Ther.

[24] Agarwal RK, Sun SH, Su SB, Chan CC, Caspi RR (2002). Pertussis toxin alters the innate and the adaptive immune responses in a pertussis-dependent model of autoimmunity. J Neuroimmunol.

[25] Su SB, Silver PB, Zhang M, Chan CC, Caspi RR (2001). Pertussis toxin inhibits induction of tissue-specific autoimmune disease by disrupting G protein-coupled signals. J Immunol.

[26] Chen X, Shao H, Zhi Y, Xiao Q, Su C, Dong L (2016). CD73 pathway contributes to the immunosuppressive ability of mesenchymal stem cells in intraocular autoimmune responses. Stem Cells Dev.

[27] Yun J, Xiao T, Zhou L, Beuerman RW, Li J, Zhao Y (2018). Local S100A8 levels correlate with recurrence of experimental autoimmune uveitis and promote pathogenic T cell activity. Invest Ophthalmol Vis Sci.

[28] Blanco P, Palucka AK, Pascual V, Banchereau J (2008). Dendritic cells and cytokines in human inflammatory and autoimmune diseases. Cytokine Growth Factor Rev.

[29] León B, Lund FE (2019). Compartmentalization of dendritic cell and T-cell interactions in the lymph node: anatomy of T-cell fate decisions. Immunol Rev.

[30] Sato N, Meijer L, Skaltsounis L, Greengard P, Brivanlou AH (2004). Maintenance of pluripotency in human and mouse embryonic stem cells through activation of Wnt signaling by a pharmacological GSK-3-specific inhibitor. Nat Med.

[31] Kerr EC, Copland DA, Dick AD, Nicholson LB (2008). The dynamics of leukocyte infiltration in experimental autoimmune uveoretinitis. Prog Retin Eye Res.

[32] Bora NS, Woon MD, Tandhasetti MT, Cirrito TP, Kaplan HJ (1997). Induction of experimental autoimmune anterior uveitis by a self-antigen: melanin complex without adjuvant. Invest Ophthalmol Vis Sci.

[33] Mochizuki M, Sugita S, Kamoi K (2013). Immunological homeostasis of the eye. Prog Retin Eye Res.

[34] Grajewski RS, Hansen AM, Agarwal RK, Kronenberg M, Sidobre S, Su SB (2008). Activation of invariant NKT cells ameliorates experimental ocular autoimmunity by a mechanism involving innate IFN-gamma production and dampening of the adaptive Th1 and Th17 responses. J Immunol.

[35] Okunuki Y, Mukai R, Nakao T, Tabor SJ, Butovsky O, Dana R (2019). Retinal microglia initiate neuroinflammation in ocular autoimmunity. Proc Natl Acad Sci USA.

[36] Jia X, Hu M, Wang C, Wang C, Zhang F, Han Q (2011). Coordinated gene expression of Th17- and Treg-associated molecules correlated with resolution of the monophasic experimental autoimmune uveitis. Mol Vis.

[37] Orme JJ, Du Y, Vanarsa K, Wu T, Satterthwaite AB, Mohan C (2016). Leukocyte beta-catenin expression is disturbed in systemic lupus erythematosus. PLoS ONE.

[38] Osman A, Yan B, Li Y, Pavelko KD, Quandt J, Saadalla A (2021). TCF-1 controls Treg cell functions that regulate inflammation, CD8^+^ T cell cytotoxicity and severity of colon cancer. Nat Immunol.

[39] Van der Veeken J, Glasner A, Zhong Y, Hu W, Wang ZM, Bou-Puerto R (2020). The transcription factor Foxp3 shapes regulatory T cell identity by tuning the activity of trans-acting intermediaries. Immunity.

[40] Wang D, Fang J, Wen S, Li Q, Wang J, Yang L (2022). A comprehensive profile of TCF1^+^ progenitor and TCF1^−^ terminally exhausted PD-1^+^ CD8^+^ T cells in head and neck squamous cell carcinoma: implications for prognosis and immunotherapy. Int J Oral Sci.

[41] Fan Y, Li Y, Yao X, Jin J, Scott A, Liu B (2022). Epithelial SOX9 drives progression and metastases of gastric adenocarcinoma by promoting immunosuppressive tumour microenvironment. Gut.

[42] Spranger S, Bao R, Gajewski TF (2015). Melanoma-intrinsic beta-catenin signalling prevents anti-tumour immunity. Nature.

[43] Karnam A, Bonam SR, Rambabu N, Wong SSW, Aimanianda V, Bayry J (2021). Wnt-beta-catenin signaling in human dendritic cells mediates regulatory T-cell responses to fungi via the PD-L1 pathway. MBio.

[44] Bak SP, Amiel E, Walters JJ, Berwin B (2008). Calreticulin requires an ancillary adjuvant for the induction of efficient cytotoxic T cell responses. Mol Immunol.

[45] Grajkowska LT, Ng D, Klinakis A, Charo IF, Jung S, Gommerman JL (2011). Notch2 receptor signaling controls functional differentiation of dendritic cells in the spleen and intestine. Immunity.

[46] Clark GJ, Silveira PA, Hogarth PM, Hart DNJ (2019). The cell surface phenotype of human dendritic cells. Semin Cell Dev Biol.

[47] Vremec D, Zorbas M, Scollay R, Saunders DJ, Ardavin CF, Wu L (1992). The surface phenotype of dendritic cells purified from mouse thymus and spleen: investigation of the CD8 expression by a subpopulation of dendritic cells. J Exp Med.

[48] Zhou H, Wang Y, Lian Q, Yang B, Ma Y, Wu X (2014). Differential IL-10 production by DCs determines the distinct adjuvant effects of LPS and PTX in EAE induction. Eur J Immunol.

[49] Hou W, Wu Y, Sun S, Shi M, Sun Y, Yang C (2003). Pertussis toxin enhances Th1 responses by stimulation of dendritic cells. J Immunol.

[50] Ronchi F, Basso C, Preite S, Reboldi A, Baumjohann D, Perlini L (2016). Experimental priming of encephalitogenic Th1/Th17 cells requires pertussis toxin-driven IL-1beta production by myeloid cells. Nat Commun.

[51] Wakatsuki A, Borrow P, Rigley K, Beverley PC (2003). Cell-surface bound pertussis toxin induces polyclonal T cell responses with high levels of interferon-gamma in the absence of interleukin-12. Eur J Immunol.

[52] Chen X, Howard OM, Oppenheim JJ (2007). Pertussis toxin by inducing IL-6 promotes the generation of IL-17-producing CD4 cells. J Immunol.

[53] Iberg CA, Bourque J, Fallahee I, Son S, Hawiger D (2022). TNF-α sculpts a maturation process in vivo by pruning tolerogenic dendritic cells. Cell Rep.

[54] Liu T, DeCostanzo AJ, Liu X, Wang H, Hallagan S, Moon RT (2001). G protein signaling from activated rat frizzled-1 to the beta-catenin-Lef-Tcf pathway. Science.

[55] Halleskog C, Schulte G (2013). Pertussis toxin-sensitive heterotrimeric G(alphai/o) proteins mediate WNT/beta-catenin and WNT/ERK1/2 signaling in mouse primary microglia stimulated with purified WNT-3A. Cell Signal.

[56] Sener AG, Afsar I (2012). Infection and autoimmune disease. Rheumatol Int.

[57] Zandman-Goddard G, Shoenfeld Y (2005). Infections and SLE. Autoimmunity.

[58] Su BY, Su CY, Yu SF, Chen CJ (2007). Incidental discovery of high systemic lupus erythematosus disease activity associated with cytomegalovirus viral activity. Med Microbiol Immunol.

[59] Sugita S, Takase H, Kawaguchi T, Taguchi C, Mochizuki M (2007). Cross-reaction between tyrosinase peptides and cytomegalovirus antigen by T cells from patients with Vogt-Koyanagi-Harada disease. Int Ophthalmol.

[60] Barzilai O, Sherer Y, Ram M, Izhaky D, Anaya JM, Shoenfeld Y (2007). Epstein-Barr virus and cytomegalovirus in autoimmune diseases: are they truly notorious? A preliminary report. Ann N Y Acad Sci.

[61] Toussirot E, Roudier J (2008). Epstein-Barr virus in autoimmune diseases. Best Pract Res Clin Rheumatol.

[62] James JA, Robertson JM (2012). Lupus and Epstein-Barr. Curr Opin Rheumatol.

[63] Silpa-archa S, Sriyuttagrai W, Foster CS (2022). Treatment for Epstein-Barr virus-associated uveitis confirmed by polymerase chain reaction: efficacy of anti-viral agents and a literature review. J Clin Virol.

[64] Landaverde JM, Danovaro-Holliday MC, Trumbo SP, Pacis-Tirso CL, Ruiz-Matus C (2010). Guillain-Barre syndrome in children aged <15 years in Latin America and the Caribbean: baseline rates in the context of the influenza A (H1N1) pandemic. J Infect Dis.

[65] Chaari A, Bahloul M, Dammak H, Nourhene G, Rekik N, Hedi C (2010). Guillain-Barre syndrome related to pandemic influenza A (H1N1) infection. Intensive Care Med.

[66] Usuki S, Taguchi K, Thompson SA, Chapman PB, Yu RK (2010). Novel anti-idiotype antibody therapy for lipooligosaccharide-induced experimental autoimmune neuritis: use relevant to Guillain-Barre syndrome. J Neurosci Res.

[67] Guilherme L, Kalil J (2010). Rheumatic fever and rheumatic heart disease: cellular mechanisms leading autoimmune reactivity and disease. J Clin Immunol.

[68] Martin E, Winn R, Nugent K (2011). Catastrophic antiphospholipid syndrome in a community-acquired methicillin-resistant *Staphylococcus aureus* infection: a review of pathogenesis with a case for molecular mimicry. Autoimmun Rev.

[69] Laudien M, Gadola SD, Podschun R, Hedderich J, Paulsen J, Reinhold-Keller E (2010). Nasal carriage of *Staphylococcus aureus* and endonasal activity in Wegeners granulomatosis as compared to rheumatoid arthritis and chronic rhinosinusitis with nasal polyps. Clin Exp Rheumatol.

[70] Huhtinen M, Laasila K, Granfors K, Puolakkainen M, Seppälä I, Laasonen L (2002). Infectious background of patients with a history of acute anterior uveitis. Ann Rheum Dis.

[71] Silva-García O, Valdez-Alarcón JJ, Baizabal-Aguirre VM (2019). Wnt/beta-catenin signaling as a molecular target by pathogenic bacteria. Front Immunol.

[72] Roy BC, Subramaniam D, Ahmed I, Jala VR, Hester CM, Greiner KA (2015). Role of bacterial infection in the epigenetic regulation of Wnt antagonist WIF1 by PRC2 protein EZH2. Oncogene.

[73] Xing Y, Zhang Y, Jia L, Xu X (2019). Lipopolysaccharide from *Escherichia coli* stimulates osteogenic differentiation of human periodontal ligament stem cells through Wnt/beta-catenin-induced TAZ elevation. Mol Oral Microbiol.

[74] Suzuki M, Mimuro H, Kiga K, Fukumatsu M, Ishijima N, Morikawa H (2009). Helicobacter pylori CagA phosphorylation-independent function in epithelial proliferation and inflammation. Cell Host Microbe.

[75] Silver PB, Chan CC, Wiggert B, Caspi RR (1999). The requirement for pertussis to induce EAU is strain-dependent: B10.RIII, but not B10.A mice, develop EAU and Th1 responses to IRBP without pertussis treatment. Invest Ophthalmol Vis Sci.

[76] Shao H, Liao T, Ke Y, Shi H, Kaplan HJ, Sun D (2006). Severe chronic experimental autoimmune uveitis (EAU) of the C57BL/6 mouse induced by adoptive transfer of IRBP1-20-specific T cells. Exp Eye Res.

[77] Shin T, Kennedy G, Gorski K, Tsuchiya H, Koseki H, Azuma M (2003). Cooperative B7–1/2 (CD80/CD86) and B7-DC costimulation of CD4^+^ T cells independent of the PD-1 receptor. J Exp Med.

[78] Odobasic D, Leech MT, Xue JR, Holdsworth SR (2008). Distinct in vivo roles of CD80 and CD86 in the effector T-cell responses inducing antigen-induced arthritis. Immunology.

[79] Cross AH, Ku G (2000). Astrocytes and central nervous system endothelial cells do not express B7–1 (CD80) or B7–2 (CD86) immunoreactivity during experimental autoimmune encephalomyelitis. J Neuroimmunol.

[80] Sugiura D, Maruhashi T, Okazaki IM, Shimizu K, Maeda TK, Takemoto T (2019). Restriction of PD-1 function by cis-PD-L1/CD80 interactions is required for optimal T cell responses. Science.

[81] Shi LL, Song J, Xiong P, Cao PP, Liao B, Ma J (2014). Disease-specific T-helper cell polarizing function of lesional dendritic cells in different types of chronic rhinosinusitis with nasal polyps. Am J Respir Crit Care Med.

[82] McGeachy MJ, Bak-Jensen KS, Chen Y, Tato CM, Blumenschein W, McClanahan T (2007). TGF-beta and IL-6 drive the production of IL-17 and IL-10 by T cells and restrain T(H)-17 cell-mediated pathology. Nat Immunol.

[83] Trinchieri G (2003). Interleukin-12 and the regulation of innate resistance and adaptive immunity. Nat Rev Immunol.

[84] Komuczki J, Tuzlak S, Friebel E, Hartwig T, Spath S, Rosenstiel P (2019). Fate-mapping of GM-CSF expression identifies a discrete subset of inflammation-driving T helper cells regulated by cytokines IL-23 and IL-1beta. Immunity.

[85] Ghoreschi K, Laurence A, Yang XP, Tato CM, McGeachy MJ, Konkel JE (2010). Generation of pathogenic T(H)17 cells in the absence of TGF-beta signalling. Nature.

[86] Lee Y, Awasthi A, Yosef N, Quintana FJ, Xiao S, Peters A (2012). Induction and molecular signature of pathogenic TH17 cells. Nat Immunol.

